# Three different polymorphisms of the *DPYD* gene associated with severe toxicity following administration of 5-FU: a case report

**DOI:** 10.1186/s13256-019-2013-z

**Published:** 2019-03-22

**Authors:** Deborah Mukherji, Sarah Abdel Massih, Arafat Tfayli, Mariam Kanso, Walid Faraj

**Affiliations:** 1 Division of Hematology-Oncology, Department of Internal Medicine, American University of Beirut, Beirut, Lebanon; 2 Department of Surgery, HPB and Liver Transplant Unit, American University of Beirut, PO Box 11-0236, Riad El Solh, Beirut, 1107 2020 Lebanon

**Keywords:** 5-FU toxicity, Compound heterozygous polymorphism, Dihydropyrimidine dehydrogenase (DPD) enzyme, DPYD gene

## Abstract

**Background:**

Dihydropyrimidine dehydrogenase deficiency secondary to polymorphisms in the *DPYD* gene can lead to significant toxicity associated with the administration of fluoropyrimidine chemotherapy.

**Case presentation:**

We report a case of a 59-year-old Lebanese woman with metastatic pancreatic cancer who received FOLFIRINOX therapy and developed severe 5-fluorouracil toxicity after a single cycle. The entire *DPYD* gene was sequenced, and the patient was found to be heterozygous for three different polymorphisms that have reportedly been associated with dihydropyrimidine dehydrogenase deficiency.

**Conclusion:**

Because data regarding the prevalence and clinical significance of several heterozygous polymorphisms in a single *DPYD* gene are very limited, we suggest that full gene sequencing should be carried out, at least in populations in which the allele frequencies are unknown.

## Introduction

Fluoropyrimidine anticancer drugs such as 5-fluorouracil (5-FU) and capecitabine are commonly prescribed for adjuvant as well as palliative treatment of various solid malignancies. Treatment with these drugs is generally well tolerated, except in a small percentage of patients who develop severe, potentially life-threatening toxicity. The clinical presentation of this toxicity is similar to a 5-FU overdose and includes myelosuppression, mucositis, stomatitis, diarrhea, skin changes, and neurological abnormalities [1]. Treatment of severe toxicity includes interruption or even discontinuation of an effective anticancer therapy and often requires hospitalization [2]. The development of toxicity causes a major health care cost and has a great impact on a patient’s prognosis owing to the limitation in the available treatment options. Although the majority of cases of severe toxicity remain unexplained, a large subset of these adverse events is associated with deficiency of the dihydropyrimidine dehydrogenase (DPD) enzyme [3]. DPD is the rate-limiting enzyme in the catabolism of the fluoropyrimidines. More than 95% of an administered dose of 5-FU is dehydrogenated in the liver by DPD and later is further modified to be eliminated in the urine [4]. This enzyme derives its importance from the facts that approximately 2 million patients receive 5-FU worldwide each year and that a deficiency can lead to death after administration of standard doses of this chemotherapeutic agent [5, 6]. Despite 0.5% and 5% of Caucasian patients being affected by total and partial deficiencies, respectively, of DPYD enzyme [6, 7], neither the U.S. Food and Drug Administration nor the European Medicines Agency currently requires pharmacogenetic testing before fluoropyrimidine administration [8]. A recent study has suggested that prospective testing for the *2A genetic variant associated with DPYD deficiency would be cost-effective [2]. We report a case of a Lebanese patient with three polymorphisms of the *DPYD* gene detected by sequencing the coding region and highly conserved exon-intron splice junctions, and we suggest that extended gene testing should be carried out, at least in populations in whom the allele frequencies are unknown.

## Case presentation

A 59-year-old Lebanese woman was started on the FOLFIRINOX chemotherapy protocol for metastatic pancreatic adenocarcinoma with irinotecan (180 mg/m^2^), 5-FU (2400 mg/m^2^), leucovorin (400 mg/m^2^), and oxaliplatin (85 mg/m^2^). She presented to the hospital 1 week after her first cycle with weight loss and decreased oral intake owing to odynophagia. She was diagnosed with grade 4 mucositis and was started on fluconazole and later acyclovir. Owing to very poor oral intake, total parenteral nutrition with electrolyte correction was necessary until the patient was able to better tolerate food. At presentation to the hospital, she also reported three or four episodes of watery bowel movements per day. All stool study results were negative, so she was considered to have grade 1 chemotherapy-induced diarrhea and was started on loperamide. On the second day of hospitalization, the patient developed febrile neutropenia, so piperacillin and tazobactam were initiated along with vancomycin. Subcutaneous filgrastim was administered daily for 9 days and then twice daily for 3 days until the neutropenia subsided. During her stay, the patient also developed a drop in hemoglobin and platelet count, as well as an erythematous rash over the trunk with desquamation of the skin under the breasts. Owing to the severe side effects, FOLFIRINOX was discontinued despite a decrease in tumor marker, and the protocol was changed to gemcitabine and nanoparticle albumin-bound paclitaxel with a good initial response to treatment. With most regimens for the treatment of advanced pancreatic cancer including 5-FU, its oral prodrug capecitabine, irinotecan, or oxaliplatin, and because of the severe reaction the patient experienced, we decided to test the patient for DPD and uridine diphosphoglucuronate-glucuronosyltransferase 1A1 (UGT1A1) deficiency. UGT1A1 deficiency is associated with Gilbert’s syndrome and increases the toxicity of irinotecan. Those two tests would help us determine which of the two drugs was the culprit and guide us to use either or both drugs in a reduced dose. The functional activity of the DPD enzyme was not measured, but the DPYD gene was analyzed by next-generation sequencing of both DNA strands of the entire coding region and highly conserved exon-intron splice junctions. The patient was found to have three different variations of the DPYD gene as follows: heterozygous pathogenic variant DPYD*2A (splice site variant 1905+1G>A, also called IVS14+1), heterozygous deficiency-associated variant c.1601G>A (p.Ser534Asn) [9, 10], and heterozygous deficiency-associated variant c.2194G>A (p.Val732Ile) (Table 1) [11, 12]. All three variations are proposed to cause a reduced activity of the DPYD enzyme. The phase of the three variants (*cis* or *trans*) could not be determined, because both parents were not available. The patient also had UGT1A1 deficiency and hence Gilbert’s syndrome.

**Table 1:** DYPD gene analysis

Nucleotide change	Amino acid change	Interpretation
c.1905+1G>A		Heterozygous pathogenic
c.1601G>A	p.Ser534Asn	Heterozygous disease-associated variant
c.2194G>A	p.Val732Ile	Heterozygous disease-associated variant

## Discussion

Genetic polymorphism in the *DPYD* gene is the most well-recognized cause of DPD deficiency, with the clinically most relevant polymorphism being DPYD*2A. The frequency of DPYD*2A is 1% to 2% in the Western world [13], and by screening for DPYD*2A, only 25% of DPD-deficient patients are identified [2]. The prevalence of the DPYD*2A polymorphism is 0.6% in the Turkish population [14], but no other studies regarding the frequency of the DPYD*2A polymorphism or other variants have been reported in the Middle Eastern or Lebanese population. When we wanted to test the patient for DPYD deficiency, two options were available: either screen for the most common variants or sequence the coding region or highly conserved exon-intron splice junctions. One meta-analysis recommended testing for the *DPYD* variants IVS14+1G>A and 2846A>T [15], and another meta-analysis recommended testing the variants c.1679 T>G and c.1236G>A/HapB3 in addition to the two previously established variants [16]. We elected to perform coding region and highly conserved exon-intron splice junction sequencing because we had no previous data reported from Lebanon, knowing that such a technique may lead to findings that are to be additionally explained, confirmed, and validated against a control population, which will open the door for further research studies. Our patient was found to have not only the DPYD*2A polymorphism but also two other deficiency-associated variants that would have been missed if the entire coding region had not been sequenced. As far as we are aware, this is the first report of three pathogenic or deficiency-associated variants in the *DPYD* gene in the same patient. One study from the United Kingdom recently identified the c.1601G>A variant to be significantly associated with fluoropyrimidine toxicity [3]. In this study, two patients were compound heterozygous for the variants c.1601G>A/c.1905+1G>A and c.1601G>A/c.2846A>T. Both experienced grade 4 toxicity requiring hospital admission for 16 and 19 days, respectively. The DPYD*2A variant may be sufficient to cause the toxicity seen, but we cannot be sure; perhaps the presence of the two other variants added to the severity of the toxicity. In addition, two of the three variants must occur on the same allele, and perhaps having two variants on the same allele contributed to a significant decrease in DPD activity. The patient received two other chemotherapeutic agents that might be partially responsible for the adverse events experienced. However, oxaliplatin toxicity generally manifests as neurological abnormalities along with liver toxicity [17]. As for irinotecan, toxicity presents mostly as severe diarrhea and myelosuppression, both of which our patient experienced [18]. The fact that the patient also has Gilbert’s syndrome may have caused irinotecan toxicity that compounded the symptoms caused by DPD deficiency. Although irinotecan has side effects similar to 5-FU, many of the side effects our patient experienced are a rare occurrence in irinotecan toxicity but are very common in 5-FU toxicity.

Had the variant DPYD*2A been tested alone and had it not been present, the toxicity would have been blamed on irinotecan alone, and 5-FU would have been used without reductions, which could have harmed the patient.

## Conclusion

New technology applied to sequencing and bioinformatics is bringing down the costs of genetic testing. Because there are very limited data regarding the prevalence and clinical significance of several heterozygous polymorphisms of the *DPYD* gene in the same individual, full gene sequencing may be considered for all patients in the future.
